# Neutrophil-to-Lymphocyte Ratio as a Marker of Diabetic Ketoacidosis Severity in Type 1 Diabetes Mellitus: A Retrospective Study

**DOI:** 10.7759/cureus.109366

**Published:** 2026-05-21

**Authors:** Rabia K Alduraibi, Omar K Alsaawi, Abdulrahman A Alkhulayfi, Naif Y Aljumaah, Ibrahim K Alharbi, Essa A Alharbi

**Affiliations:** 1 Department of Endocrine and Diabetes, King Fahad Specialist Hospital, Buraydah, SAU; 2 Department of Internal Medicine, King Fahad Specialist Hospital, Buraydah, SAU

**Keywords:** diabetic ketoacidosis, disease severity, leukocytosis, neutrophil-to-lymphocyte ratio, type 1 diabetes mellitus

## Abstract

Background: Diabetic ketoacidosis (DKA) is a serious complication of type 1 diabetes mellitus (T1DM) characterized by hyperglycemia, metabolic acidosis, and ketonemia. Early assessment of disease severity is crucial for effective management and better patient outcomes. Recent research has suggested that hematological markers, especially the neutrophil-to-lymphocyte ratio (NLR), can serve as valuable indicators of systemic inflammation and disease severity. This study aimed to evaluate the association between the NLR and DKA severity and to identify clinically relevant cutoff thresholds.

Methods: This retrospective study reviewed the records of 170 patients with T1DM and DKA admitted to King Fahad Specialist Hospital, Buraydah, Saudi Arabia, between 2023 and 2025. Demographic, clinical, and laboratory data, including complete blood counts for NLR calculations at admission, were collected. DKA severity was classified as mild, moderate, or severe based on pH and bicarbonate levels.

Results: The mean patient age was 21.7 years (standard deviation (SD): 8.74), and 88 (51.8%) of the patients were female. Moderate DKA was present in 102 (60%) of the patients, while severe DKA was present in 20 (11.8%). DKA severity was correlated with higher NLR (p=0.035), random blood glucose (p=0.001), and white blood cell count (p=0.031) and inversely correlated with pH (p<0.001) and bicarbonate level (p<0.001).

Conclusions: NLR was significantly associated with DKA severity in T1DM. Although the demographic and clinical variables lacked predictive value, an elevated NLR correlated with key metabolic abnormalities. Despite its modest diagnostic accuracy, NLR remains a practical early marker of disease severity in DKA.

## Introduction

Diabetic ketoacidosis (DKA) is a serious, potentially life-threatening complication of type 1 diabetes mellitus (T1DM), characterized by hyperglycemia, metabolic acidosis, and ketonemia. Early recognition is vital for effective treatment and improved outcomes. Recently, the neutrophil-to-lymphocyte ratio (NLR) has emerged as a simple, cost-effective inflammatory marker reflecting systemic inflammation and stress. Studies have shown the predictive value of NLR in various acute and chronic conditions, including cardiovascular diseases, infections, and metabolic disorders [1,2]. In diabetes, inflammation critically contributes to complications, making the NLR a promising tool for assessing DKA severity. However, evidence supporting its use in patients with T1DM remains limited and inconclusive.

Numerous studies have examined NLR and its potential as a marker of systemic inflammation in chronic illnesses. For instance, Imtiaz et al. pointed out that NLR could be a useful indicator of systemic inflammation, especially in Asian populations [1]. Additionally, Forget et al. provided valuable reference values for the NLR in healthy individuals, which are essential for its effective use in clinical practice [2]. A particularly important study by Scutca et al. examined pediatric cases of severe DKA and found a strong association between elevated NLR and cerebral edema. Notably, this association persisted even after accounting for factors such as age and blood parameters, suggesting that the NLR may serve as a potential predictor of neurological complications in DKA [3]. To further explore this topic, Hamza et al. examined hematological inflammatory markers in patients with T1DM. They compared patients who were uninfected with those who were suffering from DKA. Their findings indicated that patients with DKA had a higher NLR, which correlated positively with serum creatinine levels and negatively with albumin levels [4]. This suggests that NLR could be an essential indicator of both inflammation and metabolic stress in the context of DKA.

Subsequently, Aslan Sirakaya et al. examined various inflammatory markers in patients with DKA admitted to the intensive care unit (ICU). Their research revealed that patients with severe DKA had elevated NLR values that correlated with other critical biochemical factors, such as pH and bicarbonate levels. Recent research has shown a strong association between IL-6 levels and the ICU stay duration, underscoring the crucial role of inflammation in DKA [5]. While much attention has been paid to type 2 diabetes mellitus (T2DM) in diabetes research, these findings are also essential for deepening our understanding of T1DM. Chen et al. found that a higher NLR was associated with an increased risk of developing T2DM. Although T1DM and T2DM have different underlying mechanisms, they share inflammatory pathways that can affect the outcomes of conditions such as DKA [6].

Moreover, Kaya et al. revealed that NLR was higher in patients with metabolic syndrome, suggesting a common inflammatory pathway among these groups that may also apply to patients with DKA [7]. A recent meta-analysis by Qiu et al. further supported this connection, showing a link between high NLR and the incidence of metabolic syndrome [8]. This supports the notion that the NLR may serve as an indicator of cumulative metabolic stress and systemic inflammation.

Despite its potential, evidence for using the NLR as a severity marker in DKA, especially for patients with T1DM, remains limited and inconclusive. While some retrospective studies have suggested a link between elevated NLR and increased DKA severity, further research is needed to confirm these associations and to define clinically relevant cutoff values. To contribute to this area of research, this study aimed to determine the correlation between the NLR and DKA severity and establish cut-off values for clinical practice.

## Materials and methods

This was a single-center, retrospective, observational clinical study of patients with T1DM having DKA who were admitted to King Fahad Specialist Hospital, Buraydah, Saudi Arabia, between 2023 and 2025. The patients selected in the study using convenience sampling and those included were diagnosed with DKA according to the American Diabetes Association diagnostic criteria that included blood glucose levels ≥200 mg/dL, metabolic acidosis (pH <7.3, bicarbonate <18 mmol/L), and the presence of ketonemia (≥3 mmol/L) or ketonuria (ketone strip 2+ or greater) [9]. Patients with infectious diseases, medical conditions that could alter hematological parameters, other types of diabetes, hyperglycemic crisis due to causes other than DKA, such as hyperosmolar hyperglycemic state (HHS), ketosis due to causes other than DKA, such as alcoholic or starvation ketoacidosis, metabolic acidosis due to causes other than DKA, such as lactic acidosis or uremia, and incomplete hospital records were excluded from our study.

After obtaining permission from the hospital and ethics committee, data were extracted from patient records in Excel (Microsoft Corporation, Redmond, Washington, United States) for analysis. The collected data included demographics (age and sex), chronic diseases (hypertension, ischemic heart disease, heart failure, chronic obstructive pulmonary disease, chronic kidney diseases, liver diseases, and prior stroke), smoking history, clinical features (nausea, vomiting, abdominal pain, blurred vision, drowsiness, and altered mental status), vital signs, oxygen saturation, and Glasgow Coma Scale score at admission. Laboratory tests included complete blood count with differential, electrolyte, blood gas, and glycated hemoglobin (HbA1c) levels. DKA severity was classified according to the American Diabetes Association criteria by venous pH and bicarbonate: mild (pH 7.25-7.30, bicarbonate 15-18 mEq/L), moderate (pH 7.00-7.24, bicarbonate 10 to <15 mEq/L), and severe (pH <7.0, bicarbonate <10 mEq/L) [9]. NLR was calculated as the neutrophil count divided by the lymphocyte count, and platelet-to-lymphocyte ratio (PLR) was calculated as the platelet count divided by the lymphocyte count.

Statistical analysis

Data were presented as numbers and percentages for all categorical variables, whereas means and standard deviations were used for all continuous variables. DKA severity was compared with demographic, clinical, metabolic, and laboratory parameters using Fisher's exact test and one-way analysis of variance (ANOVA). Receiver operating characteristic (ROC) curve analyses were performed to determine the NLR-based cutoff points for DKA severity. Furthermore, the Pearson correlation coefficient was used to assess the relationships between NLR and age, as well as between NLR and metabolic and laboratory parameters. Statistical significance was set at p<0.05, and p<0.01 was considered highly statistically significant. All data analyses were performed using IBM SPSS Statistics for Windows, Version 26.0 (IBM Corp., Armonk, New York, United States).

## Results

In this study, 170 patients with DKA were reviewed and analyzed. As shown in Table 1, the mean patient age was 21.7 (SD 8.74). The male-to-female ratio was 82:88. The most common comorbidity seen was hypertension, which was observed in six (3.5%) of the patients. Nausea, vomiting, and abdominal pain were the most common symptoms and were observed in 134 (78.8%), 126 (74.1%), and 122 (71.8%) of the patients, respectively. The Glasgow Coma Scale classification suggested no impairment in almost all patients (169, 99.4%). In addition, 107 (62.9%) of the participants had a random blood sugar score of 500 or less. When compared to DKA severity, it was found that all demographic and clinical variables showed no significant relationships with the severity of DKA, including age, sex, comorbidities, Glasgow Coma Scale, and random blood sugar level (all p>0.05).

**Table 1:** Demographic and clinical characteristics of patients in relation to the severity of DKA (n=170) Data are presented as number (percentage). ^§^P-value has been calculated using Fisher's exact test. NA (not applicable) because X^2^ cannot be calculated for cases <5; hence, the exact significance is presented for the p-value. DKA: diabetic ketoacidosis; IHD: ischemic heart disease; CKD: chronic kidney disease; GCS: Glasgow Coma Scale; RBS: random blood sugar

Study variables	Overall, N (%) (n=170)	DKA severity			X^2^	P-value^§^
		Mild, N (%) (n=48)	Moderate, N (%) (n=102)	Severe, N (%) (n=20)		
Age in years (mean±SD)	21.7±8.74	21.4±7.74	21.9±8.73	21.6±11.2	0.051	0.950
Sex						
Male	82 (48.2%)	27 (56.3%)	45 (44.1%)	10 (50%)	1.952	0.377
Female	88 (51.8%)	21 (43.8%)	57 (55.9%)	10 (50%)		
Hypertension						
No	164 (96.5%)	46 (95.8%)	99 (97.1%)	19 (95%)	NA	0.580
Yes	6 (3.5%)	2 (4.2%)	3 (2.9%)	1 (5%)		
IHD						
No	169 (99.4%)	48 (100%)	101 (99%)	20 (100%)	NA	1.000
Yes	1 (0.6%)	0	1 (1%)	0		
CKD						
No	166 (97.6%)	46 (95.8%)	100 (98%)	20 (100%)	NA	0.755
Yes	4 (2.4%)	2 (4.2%)	2 (2%)	0		
Asthma						
No	168 (98.8%)	48 (100%)	101 (99%)	19 (95%)	NA	0.301
Yes	2 (1.2%)	0	1 (1%)	1 (5%)		
Chronic liver disease						
No	168 (98.8%)	47 (97.9%)	101 (99%)	20 (100%)	NA	0.641
Yes	2 (1.2%)	1 (2.1%)	1 (1%)	0		
Smoking						
No	165 (97.1%)	47 (97.9%)	98 (96.1%)	20 (100%)	NA	1.000
Yes	5 (2.9%)	1 (2.1%)	4 (3.9%)	0		
Nausea						
No	36 (21.2%)	12 (25%)	20 (19.6%)	4 (20%)	NA	0.732
Yes	134 (78.8%)	36 (75%)	82 (80.4%)	16 (80%)		
Vomiting						
No	44 (25.9%)	18 (37.5%)	22 (21.6%)	4 (20%)	NA	0.105
Yes	126 (74.1%)	30 (62.5%)	80 (78.4%)	16 (80%)		
Abdominal pain						
No	48 (28.2%)	15 (31.2%)	27 (26.5%)	6 (30%)	0.403	0.824
Yes	122 (71.8%)	33 (68.8%)	75 (73.5%)	14 (70%)		
Blurring vision						
No	163 (95.9%)	45 (93.8%)	99 (97.1%)	19 (95%)	NA	0.432
Yes	7 (4.1%)	3 (6.3%)	3 (2.9%)	1 (5%)		
Drowsiness						
No	152 (89.4%)	42 (87.5%)	93 (91.2%)	17 (85%)	NA	0.578
Yes	18 (10.6%)	6 (12.5%)	9 (8.8%)	3 (15%)		
GCS						
No impairment (15)	169 (99.4%)	48 (100%)	102 (100%)	19 (95%)	NA	0.118
Mild impairment (13-14)	1 (0.6%)	0	0	1 (5%)		
RBS level						
≤500	107 (62.9%)	31 (64.6%)	68 (66.7%)	8 (40%)	5.175	0.081
>500	63 (37.1%)	17 (35.4%)	34 (33.3%)	12 (60%)		

Table 2 shows the mean vital signs and metabolic and laboratory values. DKA severity correlated with higher systolic blood pressure (p=0.021), diastolic blood pressure (p=0.041), heart rate (p<0.001), anion gap (p<0.001), random blood glucose (p=0.001), white blood cells (p=0.031), and NLR (p=0.035). In contrast, DKA severity was associated with lower pH (p<0.001) and bicarbonate (p<0.001). No significant differences were found for respiratory rate, temperature, peripheral oxygen saturation, neutrophils, lymphocytes, eosinophils, monocytes, platelets, PLR, albumin, potassium, sodium, chloride, or HbA1c (p>0.05).

**Table 2:** Metabolic and laboratory parameters of the patients in relation to the severity of DKA (n=170) Data are presented as mean±standard deviation (SD). ^§^P-value has been calculated using a one-way analysis of variance (ANOVA) test. **Significant at p<0.05 level. DKA: diabetic ketoacidosis; SBP: systolic blood pressure; DBP: diastolic blood pressure; HR: heart rate; RR: respiratory rate; SpO_2_: peripheral oxygen saturation; pH: potential hydrogen; HCO_3_: bicarbonate; AG: anion gap; RBS: random blood sugar; WBC: white blood cell; NLR: neutrophil-to-lymphocyte ratio; PLR: platelet-to-lymphocyte ratio; HbA1c: glycated hemoglobin

Parameters	Overall, mean±SD (n=170)	DKA severity			F-test	P-value^§^
		Mild, mean±SD (n=48)	Moderate, mean±SD (n=102)	Severe, mean±SD (n=20)		
SBP	119.2±13.5	117.7±12.3	118.4±12.3	126.9±18.9	3.938	0.021**
DBP	73.9±9.74	74.3±9.66	72.8±9.55	78.7±9.83	3.252	0.041**
HR	98.8±16.6	95.5±15.3	97.7±15.4	112.5±19.3	8.646	<0.001**
RR	19.7±2.12	19.3±1.94	19.7±2.22	20.4±1.89	1.793	0.170
Temperature	36.9±0.27	36.9±0.26	36.9±0.26	36.8±0.29	1.878	0.156
SpO_2_	98.5±1.38	98.4±1.53	98.4±1.39	98.9±0.79	1.046	0.354
pH	7.18±0.11	7.28±0.03	7.17±0.06	6.97±0.11	161.6	<0.001**
HCO_3_	11.9±4.00	15.6±2.99	11.5±2.70	5.65±2.45	94.13	<0.001**
AG	20.6±6.57	18.7±6.41	20.3±5.59	26.4±8.47	11.05	<0.001**
RBS	468.3±152.9	433.3±169.2	463.1±135.1	578.7±155.4	6.990	0.001**
WBC	12.5±12.6	9.69±4.27	12.7±15.3	18.5±8.14	3.554	0.031**
Neutrophils	8.83±12.3	9.53±21.5	7.45±4.01	14.2±8.33	2.733	0.068
Lymphocytes	2.43±1.04	2.56±1.15	2.32±0.92	2.65±1.29	1.386	0.253
Eosinophils	0.12±0.28	0.11±0.10	0.14±0.35	0.04±0.13	0.972	0.381
Monocytes	1.29±9.32	0.59±0.29	1.70±12.0	0.80±0.48	0.259	0.772
Hemoglobin	14.3±2.17	14.3±2.00	14.5±1.78	13.5±3.76	1.783	0.171
Platelets	370.8±127.2	358.6±126.4	364.7±123.6	430.8±137.3	2.608	0.077
NLR	4.49±5.26	4.05±6.39	4.13±4.22	7.33±6.39	3.422	0.035**
PLR	181.3±105.1	162.8±77.1	183.7±108.3	213.5±138.9	1.720	0.182
Albumin	44.4±5.33	43.3±4.45	44.8±5.47	44.7±6.38	1.299	0.275
Potassium	4.64±0.71	4.52±0.58	4.64±0.65	4.94±1.12	2.504	0.085
Sodium	132.6±4.62	132.8±5.04	132.2±3.58	133.9±7.45	1.261	0.286
Chloride	100.0±6.59	98.5±6.62	100.4±5.31	101.9±10.8	2.174	0.117
HbA1c	11.5±2.69	10.7±2.48	11.8±2.53	11.6±3.66	3.025	0.051

Table 3 presents the correlations between NLR and metabolic and laboratory parameters in patients with DKA. Significant positive correlations were found between the NLR and age (r=0.446; p<0.001), systolic blood pressure (r=0.179; p=0.019), anion gap (r=0.183; p=0.017), white blood cell count (r=0.227; p=0.003), and potassium (r=0.233; p=0.002). Inverse correlations were observed with pH (r=-0.266; p<0.001) and bicarbonate (r=-0.218; p=0.004). No significant correlations were found between diastolic blood pressure, heart rate, respiratory rate, temperature, peripheral oxygen saturation, random blood sugar, eosinophils, monocytes, hemoglobin, platelets, albumin, sodium, chloride, and HbA1c levels (p>0.05).

**Table 3:** Correlation between NLR and metabolic and laboratory parameters (n=170) ^§^P-value has been calculated using a one-way analysis of variance (ANOVA) test. *Significant at p<0.05 level. **Significant at p<0.01 level. SBP: systolic blood pressure; DBP: diastolic blood pressure; HR: heart rate; RR: respiratory rate; SPO_2_: peripheral oxygen saturation; pH: potential hydrogen; HCO_3_: bicarbonate; AG: anion gap; RBS: random blood sugar; WBC: white blood cell; NLR: neutrophil-to-lymphocyte ratio; PLR: platelet-to-lymphocyte ratio; HbA1c: glycated hemoglobin

Parameters	NLR	
	r	P-value^§^
SBP	0.179	0.019*
DBP	-0.045	0.556
HR	0.035	0.650
RR	0.070	0.415
Temperature	-0.020	0.799
SPO_2_	-0.143	0.064
pH	-0.266	<0.001**
HCO_3_	-0.218	0.004**
AG	0.183	0.017*
RBS	0.093	0.230
WBC	0.227	0.003**
Eosinophils	-0.109	0.155
Monocytes	-0.021	0.790
Hemoglobin	-0.017	0.825
Platelets	-0.058	0.456
Albumin	-0.024	0.761
Potassium	0.233	0.002**
Sodium	-0.095	0.219
Chloride	-0.117	0.130
HbA1c	-0.075	0.335

**Figure 1:**
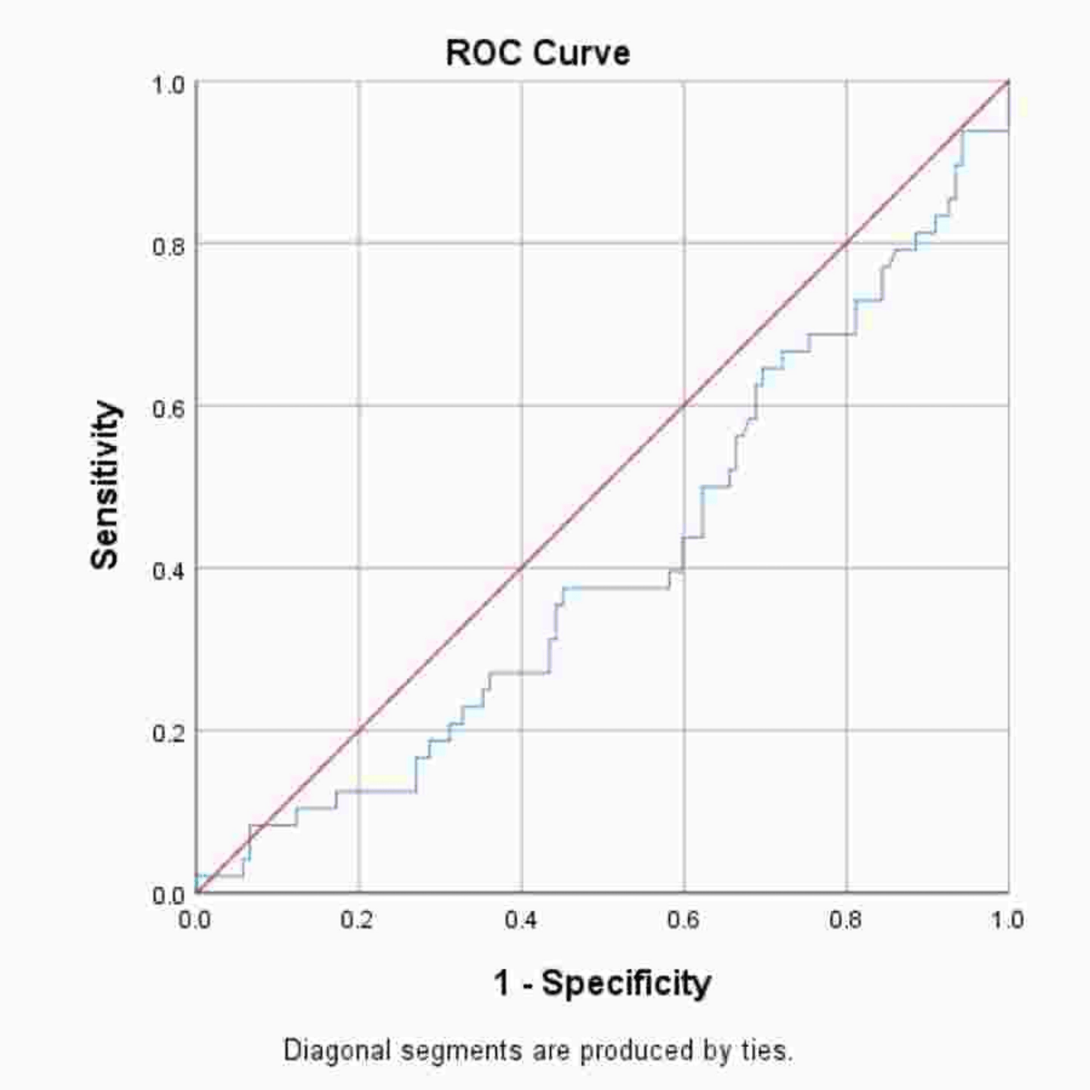
Determining the cutoff point for mild DKA using NLR DKA: diabetic ketoacidosis; NLR: neutrophil-to-lymphocyte ratio; ROC: receiver operating characteristic

Figure 1 shows that the NLR cutoff for mild DKA was 1.31, with a sensitivity of 81.3% and 1-specificity of 88.5%. The area under the curve (AUC) was 0.409.

**Figure 2:**
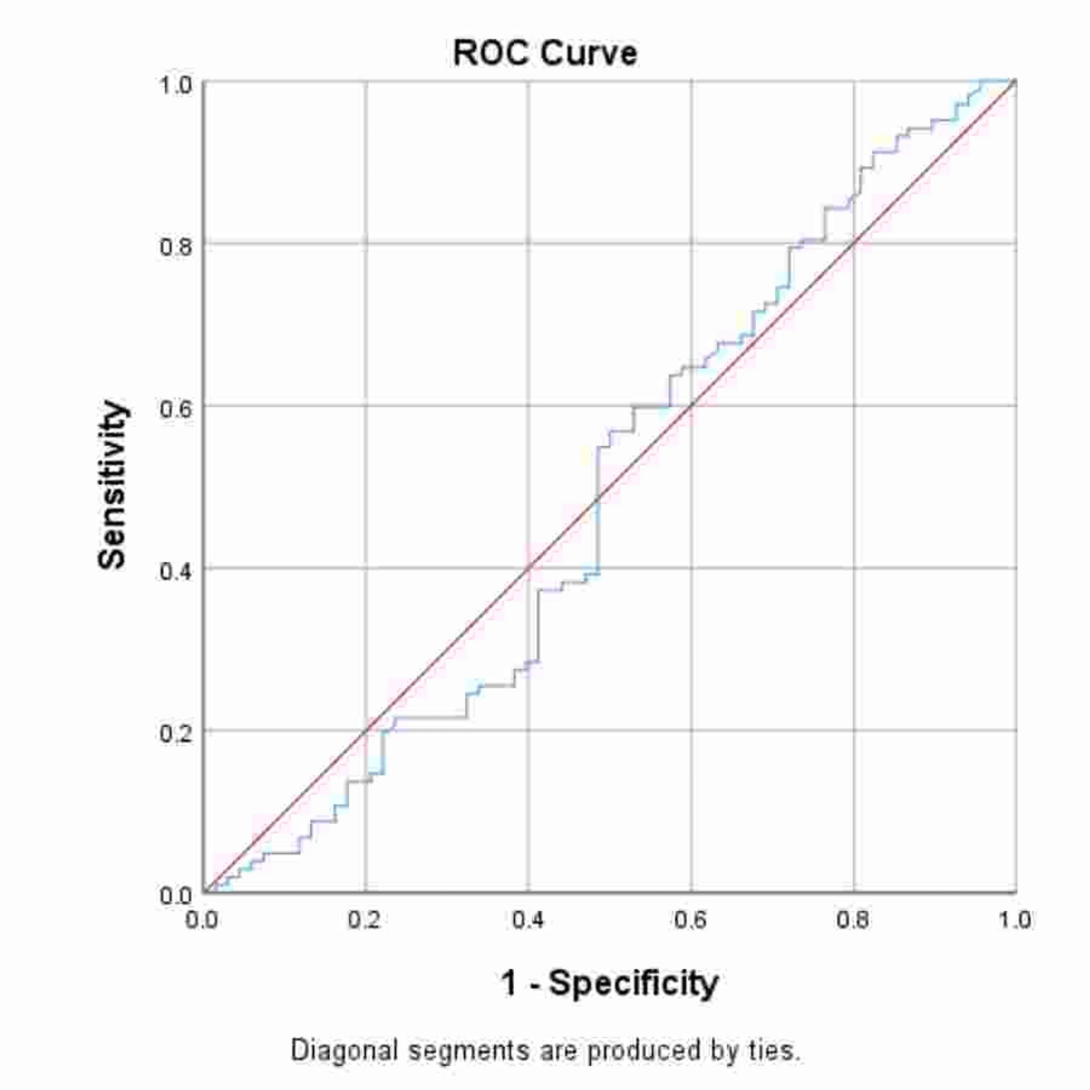
Determining the cutoff point for moderate DKA using NLR DKA: diabetic ketoacidosis; NLR: neutrophil-to-lymphocyte ratio; ROC: receiver operating characteristic

Figure 2 shows that the NLR cutoff for moderate DKA was 2.60, with a sensitivity of 57.8% and 1-specificity of 52.9%. The AUC was 0.494.

**Figure 3:**
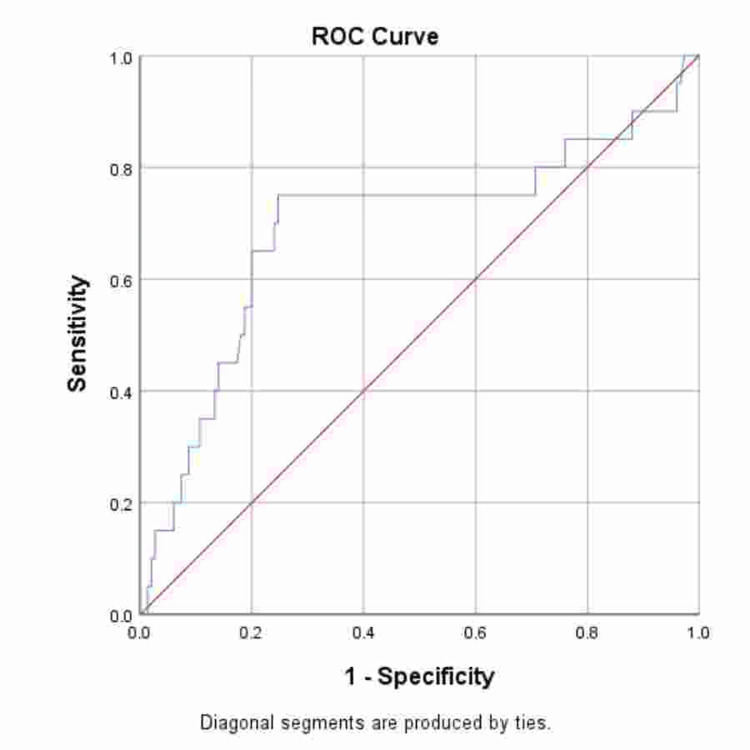
Determining the cutoff point for severe DKA using NLR DKA: diabetic ketoacidosis; NLR: neutrophil-to-lymphocyte ratio; ROC: receiver operating characteristic

Figure 3 shows that the NLR cutoff for severe DKA was 4.61, with a sensitivity of 65% and a 1-specificity of 24%. The AUC was 0.691.

## Discussion

This retrospective study, conducted at King Fahad Specialist Hospital in Buraydah, Saudi Arabia, explored the potential of NLR as a marker of disease severity in DKA among 170 patients with T1DM. Among these patients, a great proportion experienced moderate DKA, accounting for 102 (60%), while 20 (11.8) were classified as having severe DKA. Interestingly, we did not find any significant association between DKA severity and demographic or clinical factors, including age, sex, pre-existing health conditions, or presenting symptoms (all p>0.05). This finding is in contrast to the research conducted by Imtiaz et al., which indicated that NLR was elevated in patients with chronic diseases, including diabetes and hypertension. They proposed that the NLR might indicate underlying chronic systemic inflammation rather than acute metabolic crises [1]. The differences in our conclusions can be attributed to distinct research focuses. Our study focused on episodes of acute DKA. However, these studies focused on baseline inflammation in patients with stable chronic illnesses.

We found that DKA severity was significantly associated with elevated systolic blood pressure, diastolic blood pressure, heart rate, anion gap, random blood sugar, white blood cell count, and NLR and inversely associated with pH and bicarbonate. These results align with those of Aslan Sirakaya et al., who demonstrated that the NLR, IL-6, and CRP-to-albumin ratio were significantly correlated with DKA severity and ICU admission [5]. Similarly, Hamza et al. confirmed the association between the NLR and DKA severity in T1DM [4]. However, our study did not find significant correlations between respiratory rate, temperature, SPO2, or other hematological indices, which may reflect differences in sample size, timing of laboratory collection, or inclusion criteria.

The NLR cutoffs of 1.31 for mild, 2.60 for moderate, and 4.61 for severe DKA demonstrated modest discriminatory ability, with corresponding AUC values of 0.409, 0.494, and 0.691, respectively. These findings indicate that these values may not be helpful on their own for diagnosing conditions. In a study by Scutca et al., NLR was found to be a stronger predictor of cerebral edema in children with DKA, with better sensitivity and specificity [3]. The observed differences could be related to how immune responses vary with age, as well as to the fact that the NLR is used to anticipate complications rather than just to measure severity.

Our study found that the NLR was positively associated with age, systolic blood pressure, anion gap, white blood cell count, and potassium levels. However, it showed an inverse relationship with pH and bicarbonate. In a study by Cheng et al., using the NLR alongside the white blood cell count improved the ability to predict the onset of DKA in patients with T1DM without infection [10]. However, we did not find significant associations between the NLR and other factors, including neutrophils, lymphocytes, PLR, albumin, HbA1c, and other electrolytes such as sodium and chloride. Forget et al. noted that the reference values for NLR can vary across populations and clinical settings, which might help explain these differences [2].

Studies on T2DM and metabolic syndrome also support the role of the NLR as an inflammatory marker. Chen et al. and Kaya et al. found an elevated NLR in patients with poor glycemic control and metabolic syndrome [6,7]. However, these studies focused on chronic inflammation, whereas our findings reflected acute inflammatory shifts during DKA. Qiu et al.'s meta-analysis of 70,937 individuals confirmed the predictive value of NLR for metabolic syndrome; however, the context differs from that of acute DKA [8]. Additionally, Elshandalaty et al. and Elfeky et al. supported the utility of the NLR in early DKA prediction and severity assessment, particularly in pediatric and infection-free cases [11,12]. Our findings reinforce this but highlight the need for contextual interpretation and integration with other clinical markers.

This study has a few limitations that must be acknowledged. First, its retrospective design means that cause-and-effect relationships cannot be firmly established. Second, because the study was conducted at a single tertiary care center in Saudi Arabia, the results may not apply to other populations with different demographic and clinical features. Another notable aspect is that the study did not separate the patients by age, which could be significant, as inflammatory responses may differ between children and adults.

## Conclusions

Our research revealed that the NLR is closely associated with DKA severity in patients with T1DM. This suggests that NLR can serve as an indicator of acute systemic inflammation. Although demographic and clinical factors did not show predictive value, NLR, together with anion gap, white blood cell count, and pH, can be useful for evaluating DKA severity. It is essential to recognize that although NLR offers valuable insights, its reliability as an independent predictor has certain limitations. Therefore, any assessment of NLR should consider a broader clinical context and incorporate additional biochemical information. The established cutoff values can assist in early risk evaluation; however, we suggest that further studies, particularly multicenter and prospective studies, are needed to validate these results. Differences from the existing literature might arise from variations in the demographic characteristics of the study populations, study designs, timing of sample collection, and use of the NLR to anticipate the severity, complications, or overall burden of chronic disease.

## References

[1] Imtiaz F, Shafique K, Mirza SS, Ayoob Z, Vart P, Rao S (2012). Neutrophil lymphocyte ratio as a measure of systemic inflammation in prevalent chronic diseases in Asian population. Int Arch Med.

[2] Forget P, Khalifa C, Defour JP, Latinne D, Van Pel MC, De Kock M (2017). What is the normal value of the neutrophil-to-lymphocyte ratio?. BMC Res Notes.

[3] Scutca AC, Nicoară DM, Mang N, Jugănaru I, Brad GF, Mărginean O (2023). Correlation between neutrophil-to-lymphocyte ratio and cerebral edema in children with severe diabetic ketoacidosis. Biomedicines.

[4] Hamza Z, Jerbi A, HadjKacem F (2023). Neutrophil to lymphocyte ratio is associated with diabetic ketoacidosis in type 1 diabetes mellitus. Endocr Abstr.

[5] Aslan Sirakaya H, Sipahioglu H, Cetinkaya A, Aydin K (2025). Relationship between inflammatory markers (IL-6, neutrophil-lymphocyte ratio, and C-reactive protein-albumin ratio) and diabetic ketoacidosis severity: correlation with clinical outcomes. Medicina (Kaunas).

[6] Chen HL, Wu C, Cao L, Wang R, Zhang TY, He Z (2024). The association between the neutrophil-to-lymphocyte ratio and type 2 diabetes mellitus: a cross-sectional study. BMC Endocr Disord.

[7] Kaya T, Solak Y, Akçay EÜ, Ertürk Z, Ergenç H, Tamer A (2017). The relationship between neutrophil to lymphocyte ratio and metabolic syndrome in patients with type 2 diabetes. Int J Diabetes Dev Ctries.

[8] Qiu Z, Huang C, Xu C, Xu Y (2024). Predictive role of neutrophil-to-lymphocyte ratio in metabolic syndrome: meta-analysis of 70,937 individuals. BMC Endocr Disord.

[9] Umpierrez GE, Davis GM, ElSayed NA (2024). Hyperglycemic crises in adults with diabetes: a consensus report. Diabetes Care.

[10] Cheng Y, Yu W, Zhou Y, Zhang T, Chi H, Xu C (2021). Novel predictor of the occurrence of DKA in T1DM patients without infection: a combination of neutrophil/lymphocyte ratio and white blood cells. Open Life Sci.

[11] Elshandalaty HS, Alkhateeb MA, Ibrahim WS, AboElnasr SM (2024). Study of neutrophil/lymphocyte ratio as a new marker in early prediction of the occurrence of diabetic ketoacidosis in type 1 diabetes mellitus patients without infection. Egypt J Hosp Med.

[12] Elfeky NM, Ibrahim MA, Eltayeb AA, Bakry R, Hassan EA (2025). Neutrophil-to-lymphocyte ratio (NLR) and monocyte-to-lymphocyte ratio (MLR) as biomarkers in diabetic ketoacidosis. Egypt Pediatric Association Gaz.

